# Multifocal Visceral Artery Aneurysms Suggestive of Segmental Arterial Mediolysis after Living Donor Liver Transplantation: A Case Report

**DOI:** 10.70352/scrj.cr.26-0682

**Published:** 2026-11-03

**Authors:** Shunsuke Shichi, Tatsuhiko Kakisaka, Akihisa Nagatsu, Yuzuru Sakamoto, Sunao Fujiyoshi, Takeshi Aiyama, Norio Kawamura, Masaaki Watanabe, Ryoichi Goto, Naoya Kinota, Ryo Morita, Daisuke Abo, Akinobu Taketomi

**Affiliations:** 1 Department of Gastroenterological Surgery I, Hokkaido University Graduate School of Medicine, Sapporo, Hokkaido, Japan; 2 Department of Radiology, Hokkaido University Graduate School of Medicine, Sapporo, Hokkaido, Japan

**Keywords:** segmental arterial mediolysis, visceral artery aneurysm, living donor liver transplantation, Wilson disease, coil embolization, arterioportal fistula, spontaneous regression

## Abstract

**INTRODUCTION:**

Segmental arterial mediolysis (SAM) is a rare, nonatherosclerotic, and noninflammatory arteriopathy that affects medium-sized visceral arteries and can cause life-threatening aneurysms. Multifocal non-anastomotic visceral artery aneurysms are unusual after liver transplantation and may indicate an underlying arteriopathy rather than a surgical complication.

**CASE PRESENTATION:**

A 63-year-old man underwent living donor liver transplantation for decompensated cirrhosis due to Wilson disease. Preoperative imaging showed two pre-existing splenic artery aneurysms, the larger of which (20 mm) was treated by coil embolization on POD 10. Follow-up CT on POD 15 unexpectedly revealed newly apparent multifocal aneurysms involving the hepatic, gastroduodenal, right gastroepiploic, superior mesenteric, and splenic arteries, sparing the hepatic arterial anastomosis. Infection, systemic vasculitis, and anastomotic pseudoaneurysm were carefully excluded, and the multifocal, non-anastomotic distribution was considered most compatible with SAM. The untreated lesions were managed conservatively. The early course was mixed, with thrombosis of some lesions coexisting with the appearance of new aneurysms, one of which was accompanied by an arterioportal fistula and was enlarged. No rupture or bleeding from the aneurysmal lesions occurred, and by 1 year, most lesions had thrombosed or regressed, with the patient remaining well.

**CONCLUSIONS:**

SAM should be considered when rapidly emerging, multifocal, non-anastomotic visceral artery aneurysms are encountered after liver transplantation. After careful exclusion of infection, vasculitis, and anastomotic complications, conservative management with close serial imaging may be considered in selected, hemodynamically stable patients without signs of rupture. Lesions with hemodynamic factors such as an arterioportal fistula may fail to thrombose and warrant particularly close surveillance.

## Abbreviations

IgG4
: immunoglobulin G4
SAM
: segmental arterial mediolysis

## INTRODUCTION

SAM is a rare, nonatherosclerotic, and noninflammatory arteriopathy that predominantly affects medium-sized visceral arteries.^1,2)^ SAM is characterized by disruption of the arterial media and may present with various vascular abnormalities, including aneurysm formation, arterial dissection, stenosis, occlusion, intramural hematoma, and hemorrhage.^1,2)^ Although some patients are incidentally diagnosed on imaging, SAM can also cause life-threatening intra-abdominal bleeding, particularly when visceral artery aneurysms rupture.^1,3)^ The diagnosis of SAM is often challenging because its clinical and radiologic features can overlap with those of systemic vasculitis, fibromuscular dysplasia, connective tissue disorders, and infection-related aneurysms.^2)^ Histopathologic confirmation is ideal but is not always feasible in clinical practice, especially when lesions are managed conservatively or by endovascular treatment. Therefore, the diagnosis is frequently supported by a combination of characteristic imaging findings, the absence of systemic inflammatory or infectious features, and serial radiologic changes, such as spontaneous thrombosis, stabilization, or regression of untreated lesions.^2–4)^ Vascular complications after liver transplantation usually raise concern for hepatic artery thrombosis, anastomotic pseudoaneurysm, arterial stenosis, or infection-related aneurysm. In contrast, multifocal non-anastomotic visceral artery aneurysms are unusual after liver transplantation and may suggest an underlying arteriopathy rather than a local surgical complication. Splenic artery aneurysms can occur in patients with chronic liver disease and portal hypertension,^5)^ and a previous report described Wilson disease complicated by giant splenic artery aneurysms caused by fibromuscular dysplasia during living donor liver transplantation.^6)^ However, SAM-like multifocal visceral arteriopathy after liver transplantation appears to be extremely rare. Herein, we report a case of multifocal visceral artery aneurysms suggestive of SAM that became apparent after living donor liver transplantation for decompensated cirrhosis due to Wilson disease. The lesions were detected during follow-up after coil embolization of pre-existing splenic artery aneurysms and subsequently showed spontaneous thrombosis or regression during radiologic follow-up. This case highlights the importance of considering SAM in the differential diagnosis of rapidly emerging, multifocal, non-anastomotic visceral artery aneurysms after liver transplantation.

## CASE PRESENTATION

A 63-year-old man was referred to our department for living donor liver transplantation because of decompensated cirrhosis due to Wilson disease. He had previously been diagnosed with burnout nonalcoholic steatohepatitis 12 years earlier. He was subsequently diagnosed with decompensated cirrhosis due to Wilson disease based on *ATP7B* variants and a low serum ceruloplasmin level of 6.1 mg/dL. He had no history of alcohol consumption, smoking, hypertension, diabetes mellitus, dyslipidemia, atherosclerotic disease, long-term steroid use, or family history of vascular disease. His height was 167 cm, his body weight was 49.2 kg, and his BMI was 17.64 kg/m^2^. He was classified as Child–Pugh class C with a score of 11, and his Model for End-Stage Liver Disease score was 24. Preoperative CTA showed 2 splenic artery aneurysms, measuring 20 mm near the splenic hilum and 4 mm behind the pancreatic body, without apparent aneurysmal changes in the superior mesenteric artery, gastroduodenal artery, hepatic artery, or gastroepiploic artery (**Table 1**). The patient underwent living donor liver transplantation using a left lobe graft with the middle hepatic vein from his 58-year-old wife. The graft-to-recipient weight ratio was 1.19%, and the graft volume-to-standard liver volume ratio was 50.1%. Hepatic arterial reconstruction was performed between the recipient right hepatic artery and the graft left hepatic artery under microscopy. Portal vein reconstruction was performed between the recipient main portal vein and the graft left portal vein, and hepatic venous reconstruction was performed using the common trunk. Duct-to-duct biliary reconstruction was performed. The operation time was 11 hours 51 minutes, and the total blood loss, including ascites, was 21245 mL. The patient received 10920 mL of red blood cells, 10320 mL of fresh frozen plasma, and 1000 mL of platelet concentrate. During reperfusion, the systolic blood pressure transiently decreased to approximately 40 mmHg despite a maintained arterial pressure waveform, and brief manual chest compression was applied to support circulation. The blood pressure recovered within 2 minutes without administration of adrenaline. Noradrenaline and vasopressin were administered perioperatively. The patient was extubated on POD 1 and discharged from the ICU on POD 3 (**Fig. 1**). On POD 6, the patient developed melena. Upper and lower gastrointestinal endoscopy did not identify a definitive bleeding source, and small intestinal bleeding was suspected. CT showed the known splenic artery aneurysm, but no direct evidence indicated that the aneurysm was the source of the gastrointestinal bleeding. Because of the pre-existing 20-mm splenic artery aneurysm, coil embolization was performed on POD 10. Angiography confirmed the splenic artery aneurysm, which was treated by coil packing with parent artery occlusion. The procedure was technically successful, and no procedure-related complications or subsequent rebleeding from the treated aneurysm occurred (**Fig. 2**). Celiac arteriography performed during the same session also visualized the common hepatic artery and the pancreaticoduodenal arcade, neither of which showed aneurysmal change at that time; the superior mesenteric artery was not selectively injected. Follow-up CT performed on POD 15 unexpectedly revealed newly apparent multifocal visceral artery aneurysms involving the hepatic, gastroduodenal, right gastroepiploic, superior mesenteric, and splenic arteries, and as well as the splenic arterial branches (**Fig. 3**). These lesions were multifocal and non-anastomotic, and the hepatic arterial anastomosis remained patent without pseudoaneurysm formation. Repeat CT on POD 17 showed overall enlargement of the aneurysmal lesions. Because the lesions were multifocal and not suitable for additional endovascular intervention, careful observation with serial imaging was selected. The patient subsequently developed bleeding from a duodenal ulcer, which was controlled endoscopically. No extravasation from the SAM-like aneurysmal lesions was observed, and no direct communication between the ulcer and the visceral arterial aneurysms was demonstrated. Infectious aneurysms were considered unlikely because repeated blood and perioperative ascites cultures were negative, β-D-glucan remained negative, and no sustained fever, bacteremia, fungemia, or infectious focus adjacent to the aneurysmal lesions was identified. Cytomegalovirus antigenemia was negative when the aneurysmal lesions first appeared and enlarged, although transient positivity was detected later in the postoperative course. A delayed intra-abdominal abscess developed after the detection of the aneurysmal lesions; although its culture grew *Enterococcus faecium*, the abscess was anatomically separate from the aneurysmal lesions. Systemic vasculitis was also considered unlikely because there were no clinical manifestations suggestive of systemic inflammatory disease, IgG4 was within the normal range, eosinophilia was absent, and antinuclear antibody was only weakly positive at a titer of 1:40. Serial CT between PODs 30 and 56 showed a mixed course, in which gradual thrombosis of some lesions coexisted with the appearance of new aneurysms and enlargement of others (**Fig. 3**). One of the newly appearing lesions, an aneurysm of the dorsal pancreaticoduodenal arcade detected on POD 30, was accompanied by an arterioportal fistula and subsequently enlarged (**Fig. 4**). No rupture or bleeding from these lesions occurred during this period. By 1 year after transplantation, most lesions had become thrombosed or indistinct, some residual lesions had decreased in size, and no further new aneurysmal lesions had appeared (**Fig. 3**). The hepatic arterial flow and graft function remained good. The patient was alive and well 1 year after transplantation, with a performance status of 0, no recurrent bleeding, and regular outpatient follow-up.

**Table 1:** Patient characteristics and perioperative findings

**Variable**	**Findings**
Age/sex	63-year-old man
Primary disease	Decompensated cirrhosis due to Wilson disease
Wilson disease diagnosis	*ATP7B* variants and low serum ceruloplasmin level
*ATP7B* variants	dbSNP rs121907994, rs28942074, rs398123136
Serum ceruloplasmin level	6.1 mg/dL
Urinary copper level	49 microg/L; daily excretion 78 microg/day
Height/weight	167 cm/49.2 kg
BMI	17.64 kg/m^2^
Comorbidities	No hypertension, diabetes mellitus, dyslipidemia, or atherosclerotic disease
Alcohol consumption/smoking history	None/none
Long-term steroid use	None
Family history of vascular disease	None
Child–Pugh score/class	11/C
MELD score	24
Donor	Wife, 58-year-old woman
Graft	Left lobe graft with middle hepatic vein
GRWR	1.19%
GV/SLV ratio	50.1%
Hepatic arterial reconstruction	Recipient right hepatic artery to graft left hepatic artery
Number of arterial anastomoses	1
Microscope use	Yes
Portal vein reconstruction	Recipient main portal vein to graft left portal vein
Hepatic venous reconstruction	Common trunk to common trunk
Biliary reconstruction	Duct-to-duct anastomosis
Operation time	11 h 51 minutes
Blood loss	21245 mL, including ascites
Transfusion	Red blood cells, 10920 mL; fresh frozen plasma, 10320 mL; platelet concentrate, 1000 mL
Reperfusion event	Transient severe hypotension, with systolic blood pressure of approximately 40 mmHg for 2 minutes
Adrenaline use	None
Perioperative vasopressors	Noradrenaline and vasopressin
Initial immunosuppression	Tacrolimus, steroid, and mycophenolate mofetil
Anticoagulation/antiplatelet therapy	None
Perioperative antimicrobial/antifungal therapy	Ampicillin/sulbactam, micafungin, and levofloxacin
Trientine/zinc after transplantation	Discontinued after transplantation

GRWR, graft-to-recipient weight ratio; GV/SLV, graft volume-to-standard liver volume; MELD, model for end-stage liver disease

**Fig. 1:**
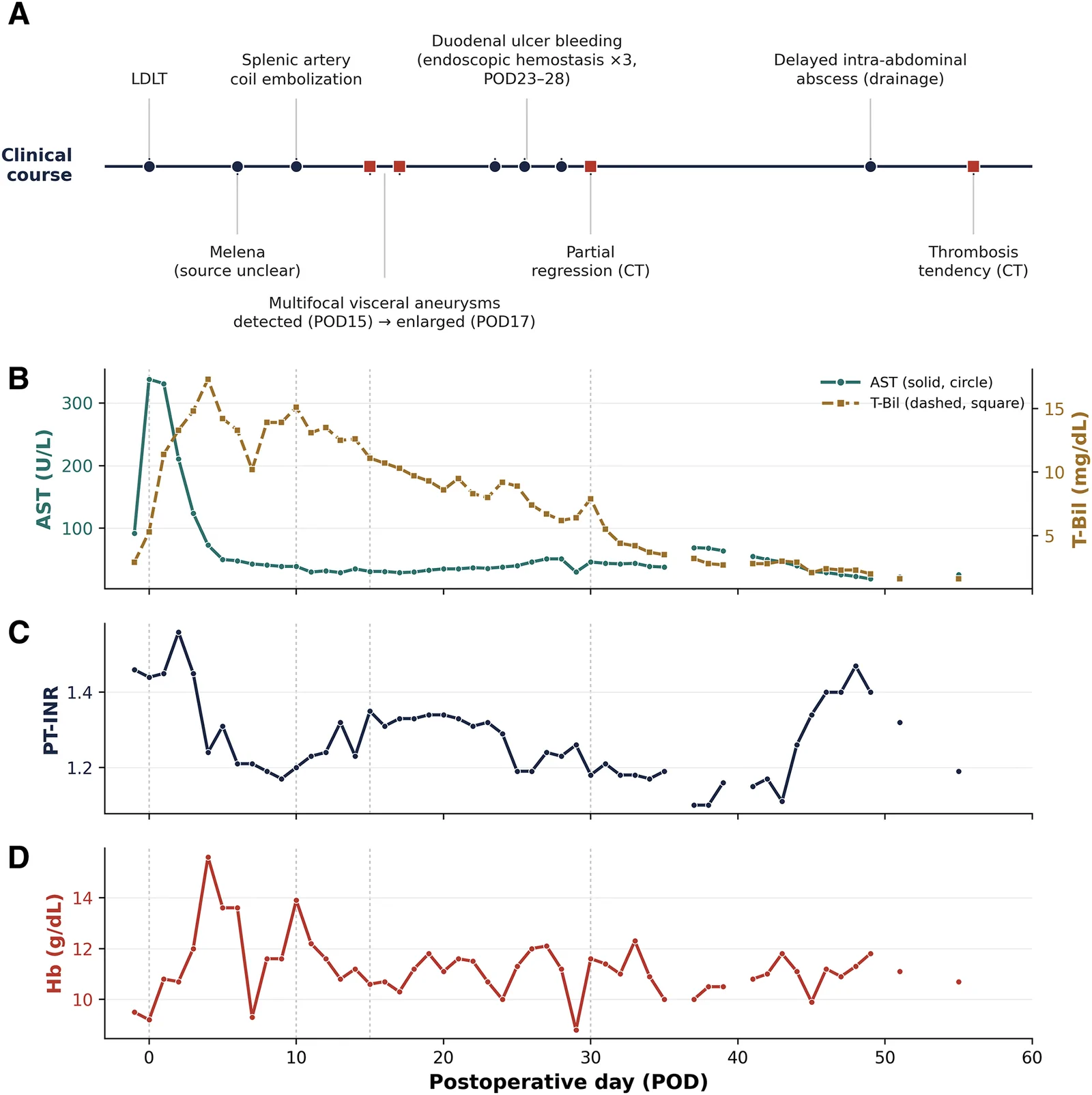
Clinical timeline and perioperative laboratory course. (**A**) Major clinical events. LDLT was performed on POD 0. A pre-existing splenic artery aneurysm was coil-embolized on POD 10, and multifocal visceral artery aneurysms became apparent on POD 15, followed by enlargement, partial regression, and thrombosis. Circles indicate general clinical events; squares indicate vascular or imaging findings. (**B**–**D**) AST and T-Bil (**B**), PT-INR (**C**), and Hb (**D**). Vertical dashed lines mark PODs 0, 10, 15, and 30. AST, aspartate aminotransferase; Hb, hemoglobin; LDLT, living donor liver transplantation; PT-INR, prothrombin time–international normalized ratio; T-Bil, total bilirubin

**Fig. 2:**
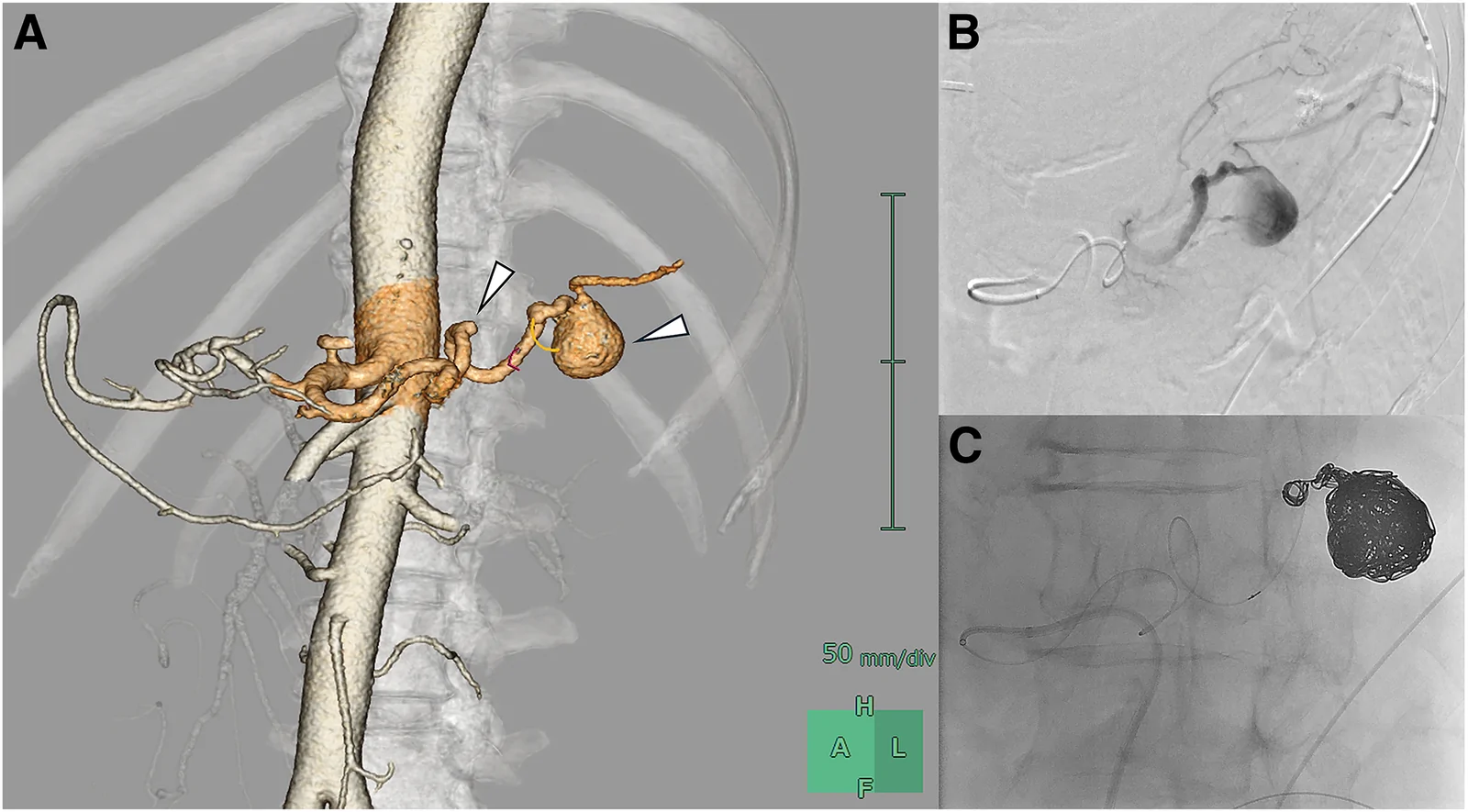
Pre-existing splenic artery aneurysms and coil embolization. (**A**) Preoperative 3D CTA shows a 20-mm splenic artery aneurysm near the splenic hilum and a 4-mm aneurysm behind the pancreatic body, without other visceral arterial abnormalities. Arrowheads indicate the 2 pre-existing splenic artery aneurysms. (**B**) Angiography confirms the 20-mm splenic artery aneurysm. (**C**) After coil embolization with parent artery occlusion, completion angiography shows no residual filling of the aneurysm.

**Fig. 3:**
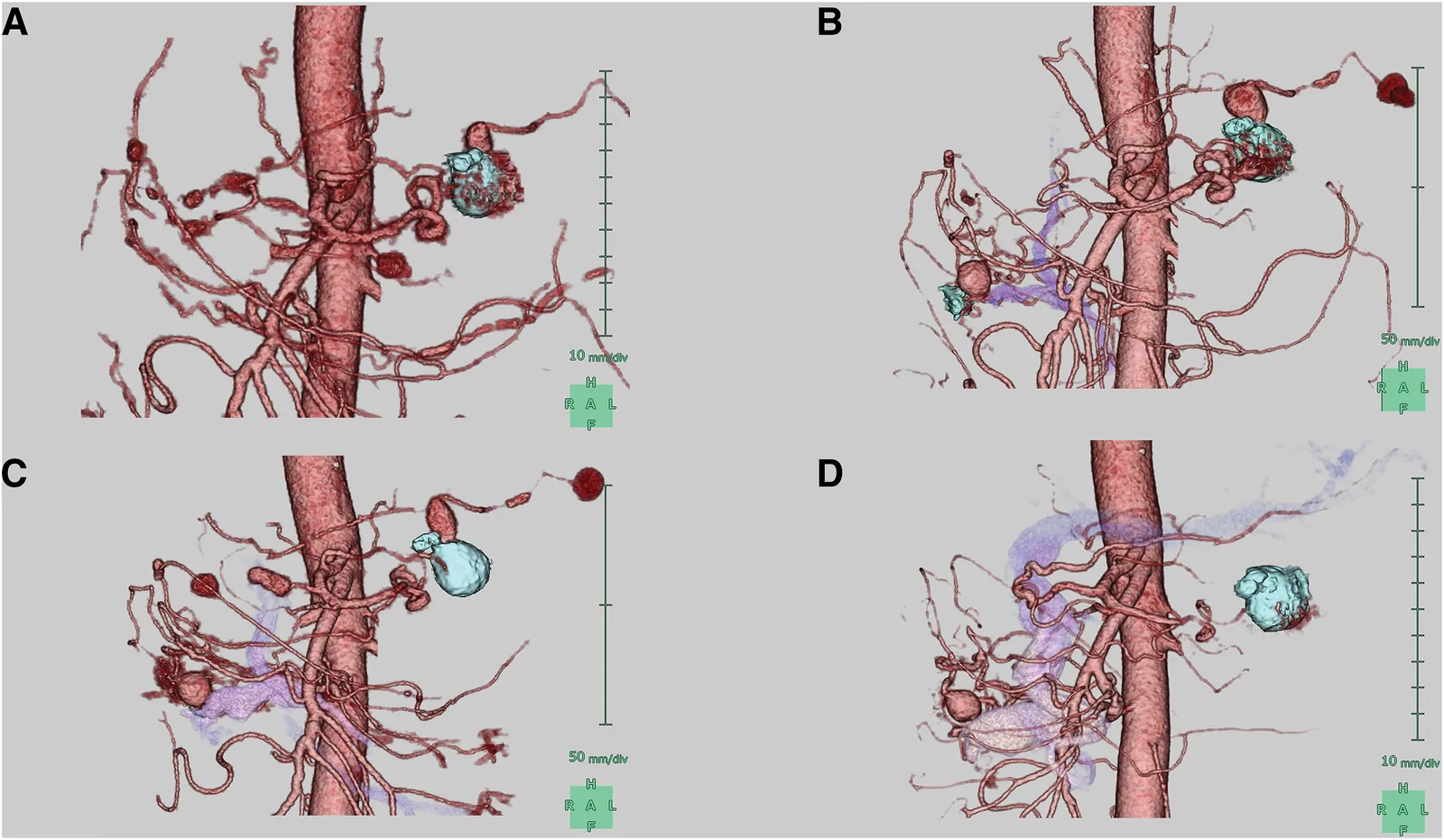
Serial 3D CTA of the multifocal visceral artery aneurysms. (**A**) At detection on POD 15, multifocal, non-anastomotic aneurysms are seen in the hepatic, gastroduodenal, right gastroepiploic, superior mesenteric, and splenic arteries and their branches, whereas the hepatic arterial anastomosis remains patent without pseudoaneurysm formation. (**B**) On POD 30, some lesions had regressed while new aneurysms had appeared, including an aneurysm of the dorsal pancreaticoduodenal arcade accompanied by an arterioportal fistula (**Fig. 4**). (**C**) On POD 56, regression of some lesions and enlargement of others coexisted. (**D**) One year after transplantation, the aneurysms were largely thrombosed, although some lesions persisted, without further new aneurysm formation.

**Fig. 4:**
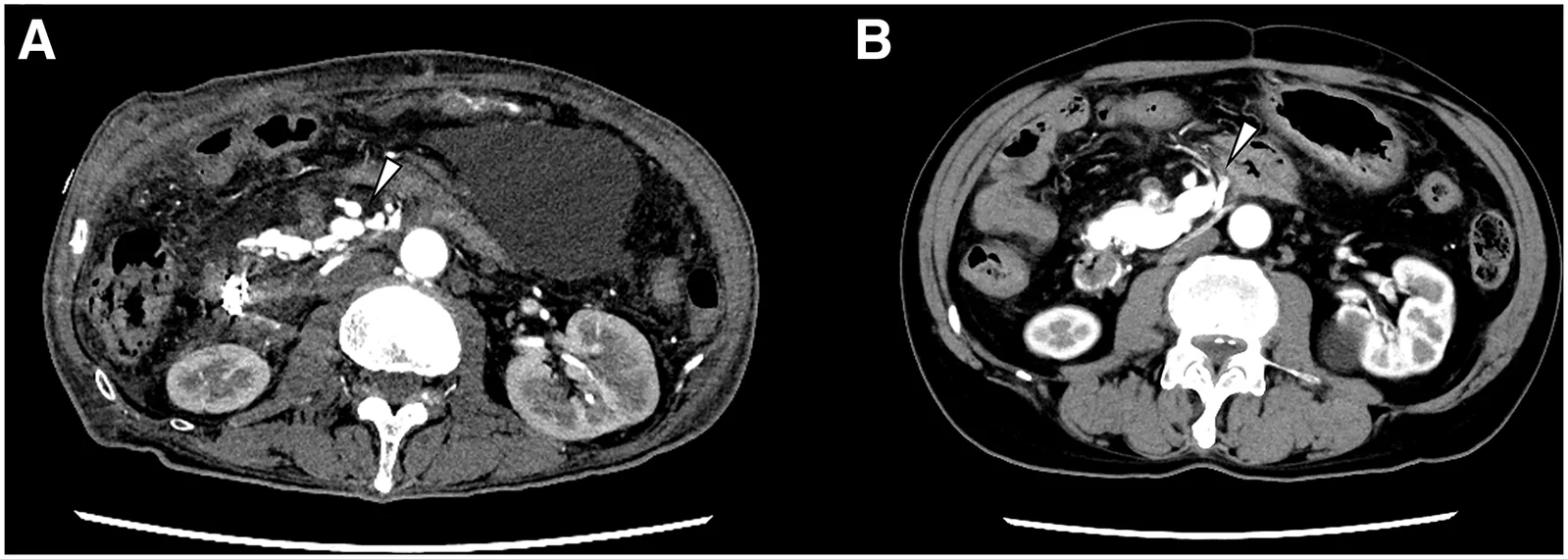
Aneurysm of the dorsal pancreaticoduodenal arcade associated with an arterioportal fistula. Axial contrast-enhanced CT in the arterial phase is shown because this lesion is difficult to evaluate on 3D reconstruction. Arrowheads indicate a saccular, beaded aneurysm of the dorsal pancreaticoduodenal arcade accompanied by an arterioportal fistula, (**A**) which newly appeared on POD 30, and (**B**) 1 year after transplantation. In contrast to the other lesions, this aneurysm did not thrombose and was enlarged.

## DISCUSSION

This case describes multifocal visceral artery aneurysms suggestive of SAM that became apparent after living donor liver transplantation. The lesions were detected during follow-up after coil embolization of pre-existing splenic artery aneurysms and involved multiple non-anastomotic visceral arteries. Although histopathologic confirmation was not available, the multifocal distribution, absence of convincing evidence for infection or systemic vasculitis, and predominant spontaneous thrombosis or regression of untreated lesions were considered most compatible with SAM-like arteriopathy. SAM is a rare, noninflammatory, and nonatherosclerotic arteriopathy that primarily affects medium-sized visceral arteries.^1,2)^ It can present with arterial dissection, aneurysm formation, stenosis, occlusion, intramural hematoma, or hemorrhage.^1,2)^ Because SAM can mimic systemic vasculitis, fibromuscular dysplasia, infection-related aneurysm, and connective tissue disorders, diagnosis requires integration of clinical, laboratory, and serial imaging findings.^2)^ In patients managed conservatively or by endovascular treatment, histopathologic confirmation is often unavailable; therefore, the absence of systemic inflammatory or infectious findings and characteristic radiologic evolution, including stabilization, thrombosis, or regression, can support the diagnosis.^3,4)^ Vascular complications after liver transplantation usually raise concern for hepatic artery thrombosis, hepatic artery stenosis, anastomotic pseudoaneurysm, or infection-related aneurysm. In the present case, however, the aneurysmal lesions were not limited to the hepatic arterial anastomosis but were distributed across multiple visceral arteries. The hepatic arterial anastomosis remained patent without pseudoaneurysm formation. Infection-related aneurysm was also considered unlikely because blood and perioperative ascites cultures were negative, β-D-glucan remained negative, and no sustained fever, bacteremia, fungemia, or infectious focus adjacent to the aneurysmal lesions was identified. Moreover, the delayed intra-abdominal abscess, although its culture grew *Enterococcus faecium*, developed after detection of the aneurysmal lesions and was anatomically separate from them, arguing against a causal infectious mechanism. These findings favored SAM-like multifocal arteriopathy over anastomotic or mycotic aneurysms. Systemic vasculitis and connective tissue disease were also considered unlikely, given the absence of clinical manifestations of systemic inflammatory disease, a normal IgG4 level, the absence of eosinophilia, and only a weakly positive antinuclear antibody titer of 1:40. Fibromuscular dysplasia could not be entirely excluded because systematic imaging of the renal, carotid, and iliac arteries was not performed. Nevertheless, several features were more consistent with SAM than with fibromuscular dysplasia. The lesions were confined to medium-sized visceral arteries and were strictly non-anastomotic, whereas fibromuscular dysplasia most often involves the renal and extracranial carotid arteries and typically presents in younger and middle-aged women, frequently with renovascular hypertension.^7)^ Our patient was an elderly man without hypertension or any clinical suggestion of renovascular or cerebrovascular disease. More importantly, the temporal behavior of the lesions was more consistent with SAM: the lesions appeared within days, evolved over weeks with concurrent regression of some lesions and emergence of others, and most untreated lesions subsequently thrombosed or regressed, whereas such rapid appearance, evolution, and spontaneous regression are not characteristic of fibromuscular dysplasia.^7)^ These observations do not exclude fibromuscular dysplasia, but taken together, they favor SAM. The mechanism by which SAM-like arteriopathy became apparent after transplantation remains uncertain. However, several perioperative factors may have contributed, including advanced liver failure, hepatorenal syndrome, intensive care management, massive blood loss and transfusion, vasopressor exposure, and transient severe hypotension during reperfusion. SAM has been proposed to be associated with vasospastic injury and catecholamine-related arterial medial damage.^1,2)^ Therefore, profound perioperative hemodynamic stress may have triggered the rapid appearance or progression of visceral arterial lesions. The presence of pre-existing splenic artery aneurysms also suggests possible underlying arterial vulnerability before transplantation. The pre-existing splenic artery aneurysms should be interpreted cautiously. Splenic artery aneurysms can occur in patients with chronic liver disease and portal hypertension, and liver transplant candidates may have splenic artery aneurysms unrelated to SAM.^5)^ Conversely, the subsequent rapid appearance of multiple non-anastomotic visceral artery aneurysms raises the possibility that the splenic artery aneurysms represented an early manifestation of an underlying arteriopathy. Importantly, preoperative CTA demonstrated no aneurysmal change or beading in the hepatic, gastroduodenal, right gastroepiploic, or superior mesenteric arteries, and celiac arteriography on POD 10 likewise showed a normal common hepatic artery and pancreaticoduodenal arcade. A multifocal arteriopathy was therefore not demonstrable either before transplantation or at the time of embolization. We accordingly interpret this case not as an arteriopathy arising de novo after transplantation, but as one in which a pre-existing arterial vulnerability became clinically and radiologically manifest and progressed rapidly under perioperative hemodynamic stress. Whether the 2 splenic artery aneurysms were themselves the earliest expression of that vulnerability or were unrelated lesions attributable to portal hypertension cannot be determined retrospectively. A previous report described Wilson disease complicated by giant splenic artery aneurysms caused by fibromuscular dysplasia during living donor liver transplantation.^6)^ Although the direct relationship between Wilson disease and SAM remains unclear, this background is relevant when interpreting the vascular findings in the present case. Management of SAM depends on symptoms, rupture risk, lesion location, and hemodynamic stability. Ruptured or symptomatic aneurysms generally require urgent endovascular or surgical treatment, whereas stable, asymptomatic, or small lesions may be managed conservatively with close imaging follow-up.^3,4,8)^ In this case, coil embolization was performed for the pre-existing 20-mm splenic artery aneurysm. After multifocal SAM-like lesions became apparent, additional intervention was not performed because the lesions were multiple and unsuitable for focal treatment. Serial CT showed that most untreated lesions eventually thrombosed or regressed without rupture or bleeding, supporting careful observation as an appropriate strategy. One lesion associated with an arterioportal fistula did not thrombose and was enlarged. Because aneurysm thrombosis depends on reduced inflow and intrasaccular stasis,^9)^ the continuous high-flow runoff into the low-pressure portal system may have prevented thrombosis and favored enlargement. This lesion arose in the pancreaticoduodenal arcade, for which repair is recommended irrespective of diameter because rupture risk is unrelated to aneurysm size.^10)^ That recommendation, however, derives largely from aneurysms associated with celiac occlusive disease, whereas SAM-like lesions may thrombose spontaneously.^3,4,8)^ In this patient, however, the risk of rupture had to be weighed against a competing risk that was specific to the early post-transplant setting. The pancreaticoduodenal arcade represents a potential collateral pathway between the superior mesenteric and celiac arterial systems. Although there was no celiac axis stenosis and the hepatic arterial anastomosis remained patent, embolization could therefore have compromised a potentially important collateral route to the graft should reconstructed hepatic arterial inflow deteriorate during the early postoperative period, when hepatic arterial flow is critical for graft and biliary viability. The aneurysms were also multiple and widely distributed, such that treating 1 lesion would not have eliminated the overall risk, and the patient was still recovering from massive intraoperative blood loss and repeated gastrointestinal bleeding. Against this background, and in the absence of rupture, extravasation, or symptoms attributable to the aneurysm, observation with close imaging surveillance was judged to carry the lower overall risk. Conservative management was not regarded as definitive. Endovascular or surgical intervention would have been considered in the event of rapid or substantial interval enlargement suggesting an increasing risk of rupture, extravasation or other signs of impending rupture, or abdominal pain or bleeding attributable to the aneurysm, with graft arterial inflow assessed beforehand and the decision made jointly with interventional radiology. The gradual enlargement observed in this lesion did not reach that threshold, and imaging surveillance was therefore continued. This case has several limitations. First, histopathologic confirmation was not obtained; therefore, the diagnosis remains radiologic and clinical. Second, fibromuscular dysplasia could not be completely excluded because systematic evaluation of the renal, carotid, and iliac arteries was not performed. Third, the causal relationship between liver transplantation, perioperative hemodynamic stress, Wilson disease, and SAM-like arteriopathy cannot be proven from a single case. Nevertheless, this case suggests that SAM should be considered when rapidly emerging, multifocal, non-anastomotic visceral artery aneurysms are encountered after liver transplantation. Because SAM may cause life-threatening rupture and 1 lesion in this patient enlarged despite observation, no general recommendation for conservative management can be drawn from a single case.

## CONCLUSIONS

SAM-like multifocal visceral arteriopathy can become apparent after living donor liver transplantation and may mimic other post-transplant vascular complications. After careful exclusion of infection, vasculitis, and anastomotic complications, conservative management with close serial imaging may be considered in selected, hemodynamically stable patients without signs of rupture, provided that the indications for intervention are repeatedly reassessed within a multidisciplinary team. Lesions with hemodynamic factors, such as an arterioportal fistula, may fail to thrombose and warrant particularly close surveillance.

## DECLARATIONS

### Availability of data and materials

The datasets supporting the conclusions of this article are included within the article.
